# Task-dependent frequency of intermuscular coherence in the presence of transcutaneous electrical spinal cord stimulation: a feasibility study

**DOI:** 10.3389/fnhum.2025.1556325

**Published:** 2025-03-10

**Authors:** Emily Lynn McNicol, Bethel Osuagwu, Aleksandra Vučković

**Affiliations:** Centre for Rehabilitation, School of Engineering, University of Glasgow, Glasgow, United Kingdom

**Keywords:** electromyography, intermuscular coherence, neuromodulation, transcutaneous electrical spinal cord stimulation, neural drive to muscles

## Abstract

The task-dependent frequency of common neural drive to muscles has important applications for motor rehabilitation therapies. While it is well established that muscle dynamics influence the synchronicity of neural drive, the modulation of this coherence between static and dynamic movements remains unclear. Transcutaneous electrical spinal cord stimulation (TESCS) is believed to enhance spinal cord excitability, potentially improving brain-muscle communication; however, its effect on common neural drive to muscles has not yet been reported. This study aimed to investigate differences in intermuscular coherence (IMC) frequency between static and dynamic movement tasks and determine whether it is feasible to enhance it by TESCS. Participants performed static and dynamic hand grip tasks at different timepoints with respect to stimulation, set to 80% tolerable intensity. Surface EMG signals were recorded from the *flexor digitorum superficialis* (FDS) and *extensor digitorum communis* (EDC) muscles during each trial to determine beta- (15–30 Hz) and gamma- (30–48 Hz) band intermuscular coherence. The sum of IMC (*IMC*_*area*_) was significantly greater (*p*_*B*_ = 0.018, *p*_*D*_ = 0.0183, *p*_*IM*_ = 0.0172, *p*_5_ = 0.0206, *p*_10_ = 0.0183, *p*_15_ = 0.0172) in the gamma-band for the dynamic task compared to the static task at every timepoint (before TESCS, during TESCS and immediately, 5-min, 10-min, and 15-min after TESCS) which may reflect a mechanism of increased efficiency of corticospinal interactions and could have implications for the types of movements that should be performed while receiving TESCS. There was no immediate measurable effect of TESCS on *IMC*_*area*_ at any timepoint in the beta-band (*p* = 0.25, *p* = 0.31) or gamma-band (*p* = 0.52, *p* = 0.73) for either the static or dynamic task respectively. This could be explained by corticospinal networks already working at maximum capacity in able-bodied individuals or that a longer duration of TESCS is required to elicit a measurable effect. While the intra-task difference in beta- and gamma-band *IMC*_*area*_ between static and dynamic tasks was statistically significant (*p*_*IM*_ = 0.0275, *p*_5_ = 0.0275, *p*_15_ = 0.0031) at timepoints after stimulation, we did not find direct evidence that TESCS influenced this beta-gamma interaction. Thus, further investigation is needed to establish any causal relationship.

## 1 Introduction

Neural drive to muscle refers to the neural activation signal the muscle receives from the pool of innervating motor neurons, generated by the transmission of common oscillatory inputs into motor neuron output (Farina et al., 2013, 2014; Dideriksen and Farina, 2019). Intermuscular coherence (IMC) analysis can be used to investigate this neural drive by evaluating the synchronistic neural input to motor units of two separate muscles. Changes in IMC observed in physiologically relevant frequency bands (alpha, beta, and gamma) can provide information on the organization of spinal pathways responsible for volitional motor output (Zipser-Mohammadzada et al., 2022; Nishimura et al., 2009). IMC in lower, alpha, frequency bands are thought to originate subcortically. Previous studies have highlighted the prominence of alpha-band IMC in people with complete spinal cord injury (SCI), suggesting coherence in this frequency band is of spinal origin (Norton et al., 2003, 2004; Norton and Gorassini, 2006). Whereas IMC in beta and gamma frequency bands are associated with cortical activation, supported by studies demonstrating significantly reduced coherence across these frequencies following spinal cord injury (Norton and Gorassini, 2006; Hansen et al., 2005; Zipser-Mohammadzada et al., 2022). Thus, it is thought that coherence in beta- and gamma- bands represent common cortical drive to muscles and can provide insight into the integrity of descending pathways (Zipser-Mohammadzada et al., 2022; Norton and Gorassini, 2006). This was demonstrated in a study by Norton and Grossini that found increased high frequency (24–40Hz) IMC between antagonistic hamstring and *vastus lateralis* muscles during walking in SCI individuals following locomotor training. This increase in IMC was associated with increased maximum motor evoked potential (MEP) amplitude, suggesting improved communication of descending pathways, resulting in greater motor unit synchronization (Norton and Gorassini, 2006). Similarly, a study by Bravo-Esteban et al. (2014) observed greater beta-band intra-muscular tibialis anterior coherence during isometric contraction in individuals with less severe SCI and attributed this to the integrity of the corticospinal pathway, resulting in greater motor unit synchronization.

It is widely accepted that beta- and gamma-band IMC reflect cortical descending drive however, muscle dynamics may influence the prominence of IMC in these frequency bands. Despite substantial overlap between the neuronal mechanisms of IMC in these frequency bands, beta-band IMC is most prominently observed during static muscle contraction, whereas IMC in the gamma-band is related to dynamic movements (Kenville et al., 2020; Gwin and Ferris, 2012; Bravo-Esteban et al., 2017). Although this movement-dependent distinction in frequency of IMC is well established, the change in magnitude of beta-band IMC between static and dynamic movements is less clear. There is evidence that beta-band IMC diminishes during dynamic movements (Kilner et al., 1999; Omlor et al., 2007) however, conflicting results also exist (Kenville et al., 2020; Reyes et al., 2017). Consolidating how neuronal coordination varies between tasks may deepen our understanding of motor control mechanisms which has important applications in SCI rehabilitation.

Transcutaneous electrical spinal cord stimulation (TESCS) is a neuromodulatory technique that involves stimulating spinal networks via a non-invasive cathode electrode placed on the skin over the spinal cord. It is suggested that stimulation at sub motor threshold level strengthens descending pathways, by activating afferent neurons within dorsal roots, increasing spinal cord excitability (Levins and Moritz, 2017; Harkema et al., 2011). This increase in excitability is thought to facilitate the activation of motor neurons. Indeed, studies have demonstrated the potential of TESCS for the restoration of upper and lower limb volitional control in people with spinal cord injury (Zhang et al., 2020; Inanici et al., 2018; Gad et al., 2018; McHugh et al., 2020; Sayenko et al., 2019; Gerasimenko Y. P. et al., 2015). A recent study by Balaguer et al. has further expanded our understanding of the neural mechanisms contributing to volitional motor control during sub threshold spinal cord stimulation. This study demonstrates the critical role of supraspinal inputs during stimulation in facilitating excitatory postsynaptic potentials, which are transformed into suprathreshold events, enabling volitional motor control (Balaguer et al., 2023).

Since these underlying neural mechanisms of TESCS are postulated to enhance the efficiency of the communication pathway between the brain and muscle (Taylor et al., 2021; McHugh et al., 2020), it is of interest whether this could enhance the task-dependent distinction in frequency of IMC. Although previous studies have demonstrated increased cortical MEP amplitude following TESCS in able-bodied individuals, reflecting increased corticospinal excitability, the effect of TESCS on coherence has not yet been reported (Megía-García et al., 2020; Kumru et al., 2021). This would provide insight into the underlying mechanisms of TESCS underpinning motor recovery in SCI. Furthermore, since TESCS is suggested to be more effective when delivered with physical therapy, analyzing the effect of TESCS on IMC under static and dynamic force outputs may provide important information on the types of movements that could enhance the functional outcomes of TESCS (Inanici et al., 2018).

The aim of this study was to consolidate the observed distinction in the frequency of IMC between static and dynamic movement tasks and determine whether it is feasible to further amplify this difference using TESCS. Based on previous evidence, it was hypothesized that IMC would be greatest in the beta-band during static contraction and the gamma-band during dynamic contraction. Furthermore, given reports of improved functional outcomes and increased excitability, TESCS is expected to facilitate motor unit synchronization, emphasizing this distinction in frequency of IMC between movement tasks.

## 2 Methods

### 2.1 Participants

Twenty adult, able-bodied volunteers (11 females and 9 males; 26.7 ± 4.8 years old) participated in this study. Participants had no history of neurological injuries or diseases and were not taking any medication that influences neural excitability (antidepressants, antipsychotics, antiepileptic).

Ethical approval was obtained from the College of Science and Engineering Ethical Committee. The study was conducted according to the Declaration of Helsinki and written informed consent was obtained from all participants.

### 2.2 Experimental protocol

**Figure 1:**
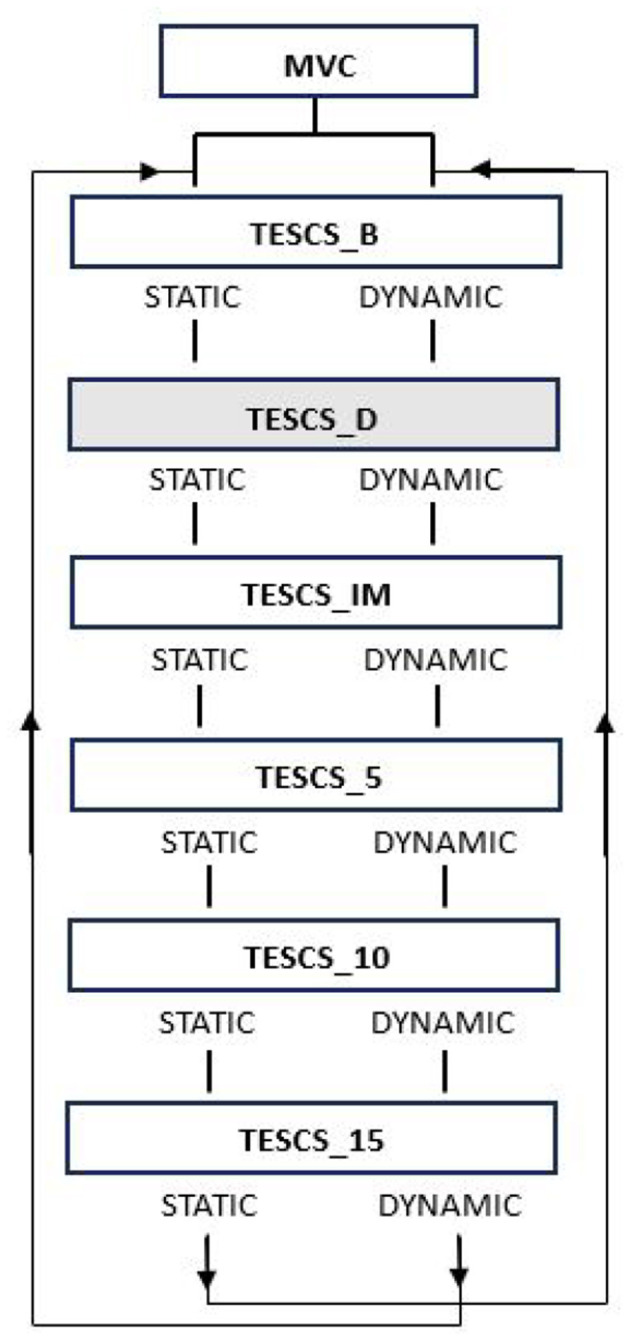
Study order of events. Maximum voluntary contraction (MVC) was performed at the start of the session. Participants then performed two different hand grip tasks at six timepoints: before TESCS (TESCS_B); during TESCS, set to 80% tolerable intensity (TESCS_D); and immediately (TESCS_IM), 5 min (TESCS_5), 10 min (TESCS_10), and 15 min (TESCS_15) after TESCS. Shading indicates application of TESCS.

Participants completed the Edinburgh Handedness Inventory questionnaire at the beginning of the session to identify their degree of handedness (Oldfield, 1971). From which, all participants were deemed to be right hand dominant. Subsequently, participants performed both static and dynamic hand grip tasks with their dominant limb which followed the same procedure, illustrated in Figure 1. Both the static and dynamic condition consisted of 6 trials at different timepoints: before TESCS (TESCS_B); during TESCS, set to 80% tolerable intensity (TESCS_D); and immediately (TESCS_IM), 5 min (TESCS_5), 10 min (TESCS_10), and 15 min (TESCS_15) after TESCS.

**Figure 2:**
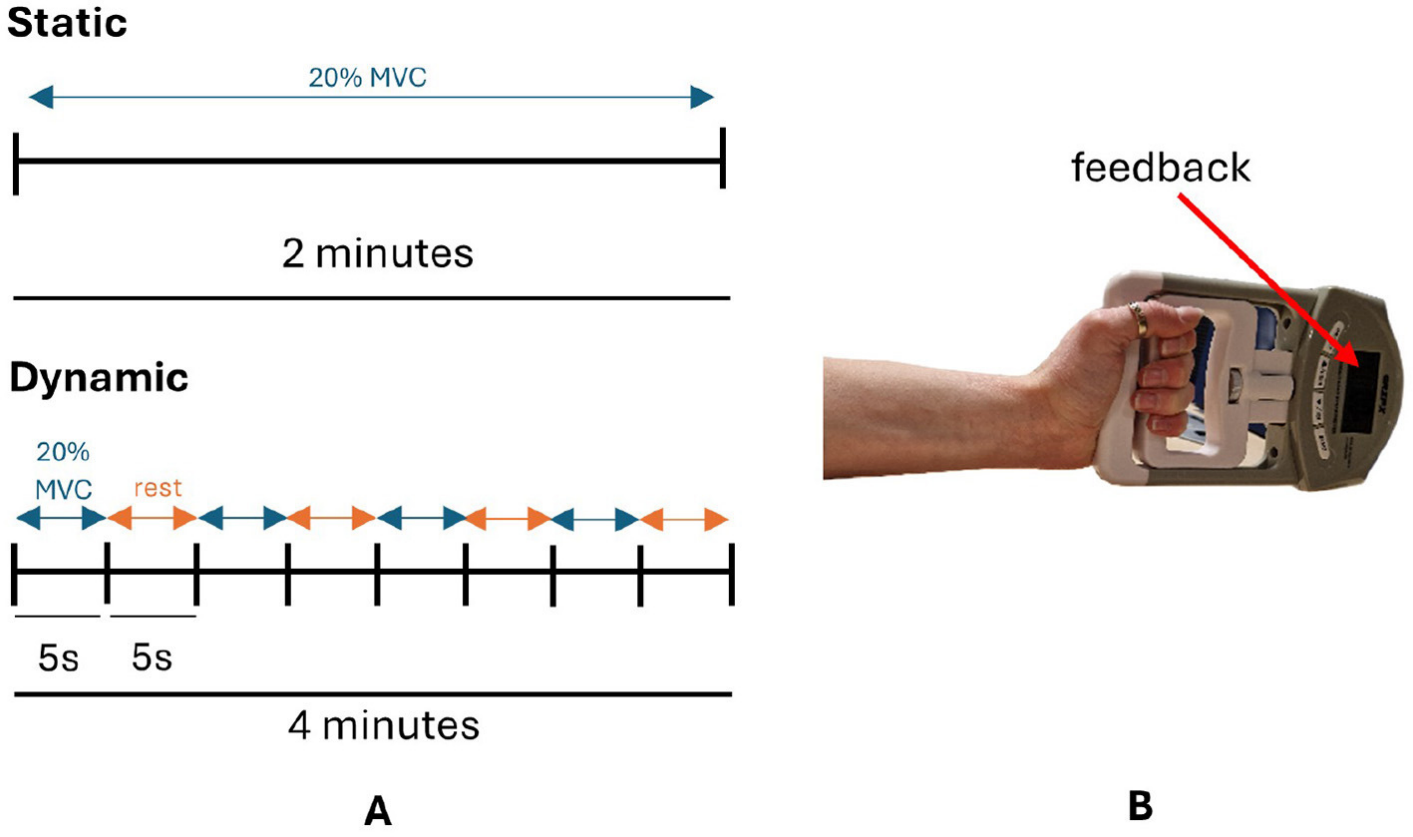
**(A)** Schematic diagram of static and dynamic tasks. **(B)** Participants maintained a cylindrical grip of the dynamometer with their wrist in a neutral position during hand grip tasks. MVC, maximum voluntary contraction.

For the static condition, participants maintained 20% maximum voluntary contraction (MVC) with a handheld dynamometer for trials of 2 min, using the force level displayed on the dynamometer screen as feedback, as detailed in Figure 2. The dynamic condition involved cycles of gripping the dynamometer to 20% MVC for 5 seconds followed by 5 seconds rest until approximately 2 min of data was collected, illustrated in Figure 2, again using the force level displayed on the dynamometer screen as feedback. Participants were instructed to maintain a neutral wrist position and 90° elbow flexion during hand grip tasks. The order in which participants performed the static and dynamic condition was randomized: group A performed the static condition first followed by the dynamic condition and vice versa for group B. Both conditions were performed consecutively on the same day. Participants were instructed to maintain a neutral wrist position and the elbow at 90° flexion during hand grip trials. At the beginning of the session participants performed 3 trials of maximum voluntary contraction for 3 seconds, from which 20% MVC was calculated.

### 2.3 Transcutaneous electrical spinal cord stimulation

A constant current stimulator (DS8R; Digitimer, Oxford, United Kingdom) delivered stimulation in 40μs biphasic rectangular pulses at a frequency of 30Hz with each pulse filled with a carrier frequency of 10kHz. This stimulation paradigm is believed to provide more efficient current transmission to the spinal cord by minimizing skin capacitance, while suppressing the activation of pain receptors (Ward, 2009; Gerasimenko et al., 2015b; Manson et al., 2020). A round cathode electrode (3.2 cm; Axelgaard Manufacturing Co, Fallbrook, CA, USA) was positioned between the C3-C4 spinous processes and secured with hypoallergenic tape to ensure sufficient contact with the skin throughout the session. Two rectangular anode electrodes (5 × 9cm; Axelgaard Manufacturing Co, Fallbrook, CA, USA) were placed symmetrically over the iliac crests. Setting the stimulation intensity based on participant tolerance is a commonly used approach (McGeady et al., 2021, 2022; McHugh et al., 2020; Samejima et al., 2022; Gad et al., 2018). To ensure participant comfort, safety, and adherence to the study protocol, it was deemed ethically appropriate to use 80% of the maximum tolerable intensity. At the beginning of the session the participants' tolerable intensity was determined by gradually increasing the intensity until the participant deemed it too uncomfortable or at a level that they would not be able to tolerate for more than 5 min (McGeady et al., 2022). The stimulation intensity was set to 80% of the participants' tolerable intensity (37.9 ± 13.0mA, range: 14.9–60.0mA) for the duration of the spinal stimulation trials of the static and dynamic condition (TESCS_D), which lasted approximately 2 and 4 min respectively. Although it has been shown that 10–20 min of TESCS can induce neuromodulation in able-bodied individuals (Megía-García et al., 2020; Benavides et al., 2020), this duration was deemed unsuitable for the present study to prevent carry over effects between conditions, which had to be conducted on the same day to maintain consistent electrode positioning for reliable comparison. Thus, this study focuses on the immediate effect of TESCS assessed before, during and immediately after stimulation. This focus is relevant as studies have demonstrated that the functional abilities of people with SCI improve while receiving stimulation (Moritz et al., 2024; Inanici et al., 2021, 2018), offering the benefit of enhanced participation in rehabilitation therapy. Therefore, if TESCS has an immediate effect as believed, neuromodulation should be observable without a 10–20 min application period.

### 2.4 EMG recordings

Surface electromyography (EMG) electrodes (Biometrics LTD surface EMG sensors SX230, UK) were placed on the muscle belly of the *biceps brachii* (BB), *flexor digitorum superficialis* (FDS), and *extensor digitorum communis* (EDC) of the right hand, as tasks were performed with the right hand since this was the dominant limb of all participants based on the Edinburgh Handedness Inventory questionnaire previously mentioned. EMG signals were recorded using a DataLINK amplifier (Biometrics LTD, UK) at a sampling rate of 1,000 Hz with internal band-pass filter of 20–450 Hz. Offline, powerline noise and stimulation artifacts were filtered out using a notch filter based on discrete Fourier transform (DFT).

There is debate as to whether rectifying EMG signals is an appropriate pre-processing step for coherence analysis. It has been shown that rectification amplifies the power of lower frequency components and better reflects the neuronal firing rate of motor units (Halliday et al., 1995; Stegeman et al., 2010; Myers et al., 2003). However, studies have since clarified the valid application of EMG rectification for purposes of coherence analysis is dependent on the level of amplitude cancellation, with only low levels of contraction requiring this pre-processing step (Farina et al., 2013; Dideriksen and Farina, 2019). Diderisken and Farina reported the correlation between rectified EMG signals and neural drive decreases linearly with amplitude cancellation, concluding that rectified EMG of contraction levels above 15% MVC do not reflect neural drive (Dideriksen and Farina, 2019). Furthermore, it is highlighted that EMG rectification should not be used when assessing common neuronal input using coherence methods across different conditions which vary amplitude cancellation (Farina et al., 2013; Dideriksen and Farina, 2019). On the contrary, raw EMG signals are not influenced by cancellation and are therefore less affected by these factors (Farina et al., 2013). Since this study evaluates coherence estimates of moderate muscle activity (20% MVC) for comparison across different movement tasks which will differ in amplitude cancellation, EMG rectification was considered inappropriate for this analysis.

### 2.5 Data analysis and statistical evaluation

Intermuscular coherence (IMC) between the FDS and EDC was calculated, as this antagonist muscle pair co-contracts during hand grip (Motawar et al., 2016; Charissou et al., 2017; Forman et al., 2019). Coherence was calculated before, during and after active stimulation (TESCS_B, TESCS_D, TESCS_IM, TESCS_5, TESCS_10, and TESCS_15) for both conditions (static and dynamic). Data recorded during the dynamic task were manually epoched per 5 second contraction period and concatenated before performing coherence analysis, ensuring methodological consistency and direct comparability between conditions. Coherence and EMG power spectral density analysis was performed using MATLAB NeuroSpec version 2.0 scripts (www.neurospec.org), employing Equation 1 for coherence estimates. Where *S*_*xy*_ is the cross-spectrum of two signals (x and y) normalized by the power spectra of signal *x*, *S*_*xx*_, and signal *y*, *S*_*yy*_ (Halliday et al., 1995). Data was segmented into *L* non-overlapping segments for coherence analysis and then averaged to get the final spectral estimates. Given the sampling rate (1,000 Hz), the segment length (*T*) was set to 1024 data points (a power of 2), and each segment was analyzed with the same DFT segment length (*S=T*) for efficient spectral estimation. This resulted in *L* = 117 complete non-overlapping segments, calculated by dividing the total length of the recording (120,000 data points) by the segment length (1,024 points). Only complete segments were included in the analysis; any data points at the end of the recording that do not form a complete segment were excluded.


(1) $$\begin{array}{l} \;C\left(\omega \right)=\frac{\left|S_{xy}{\left(\omega \right)}^{2}\right|}{S_{xx}\left(\omega \right)S_{yy}\left(\omega \right)} \end{array}$$

Significant IMC estimates, above the 95% confidence limit, were summed across frequency bands of interest, 15-30Hz and 30–48 Hz, for each participant and pooled for each timepoint (TESCS_B, TESCS_D, TESCS_IM, TESCS_5, TESCS_10, and TESCS_15) and condition (static and dynamic). These frequency bands encompass the range of frequencies previously measured for coherence analysis of static and dynamic forces, allowing comparison with other studies (Omlor et al., 2007; Gwin and Ferris, 2012; Marsden et al., 2000; Kenville et al., 2020). Although alpha-band IMC would be of interest due to its spinal origin, this band was omitted due to the 20Hz cut off frequency of the internal EMG bandpass filter. It should be noted that there is an overlap between the frequency bands of interest and the notch filter used to remove the stimulation artifact. To minimize the disadvantages of this filtering, a procedure similar to that of McGeady et al. was followed, implementing a narrow 1 Hz bandwidth (McGeady et al., 2021). Only coherence estimates above the 95% confidence limit were considered as coherence estimates lying below this limit indicate a lack of linear association between the two signals where zero coherence is considered plausible at that frequency (Halliday et al., 1995). Confidence limits were defined by Equation 2, where L is the number of non-overlapping disjoint segments used to estimate the spectra (Halliday et al., 1995). Coherence was analyzed as the sum of IMC (*IMC*_*area*_) across frequency bands of interest, as this is deemed to be more physiologically relevant than measuring coherence peaks (Kenville et al., 2020; Spedden et al., 2019). Data was subsequently normalized over the frequency band, using Equations 3, 4 for beta- and gamma-bands respectively, before conducting statistical tests. Where *A*_*coh*_(β) is the normalized sum of IMC across the beta-band, 15–30 Hz, and *A*_*coh*_(γ) is the normalized sum of IMC across the gamma-band, 30–48 Hz.


(2) $$\begin{array}{l} \;CI=1-{\left(0.05\right)}^{\frac{1}{L-1}} \end{array}$$

(3) $$\begin{array}{l} A_{coh}\left(\beta \right)=\frac{\sum_{f=15}^{30}IMC}{15} \end{array}$$

(4) $$\begin{array}{l} A_{coh}\left(\gamma \right)=\frac{\sum_{f=30}^{48}IMC}{18} \end{array}$$

A Shapiro-Wilk test was performed to evaluate the normality of the data. As normality assumptions were not satisfied, a Friedman test was carried out to determine the effect of timepoint on *IMC*_*area*_. Wilcoxon signed-rank tests were performed to determine the difference in *IMC*_*area*_ between frequency bands and to identify the effect of contraction type (static and dynamic) on *IMC*_*area*_. The p-values were adjusted using the Benjamini-Hochberg (BH) correction method where appropriate. False discovery rate (FDR) controls for the proportion of type I errors (incorrect rejections of the null hypothesis).

To demonstrate the intra-task relation in magnitude between beta- and gamma-band *IMC*_*area*_ for static and dynamic conditions, the difference in *IMC*_*area*_ between normalized beta- and gamma-bands was calculated for each timepoint using Equation 5 (Omlor et al., 2007). Where *A*_*c*_ is the intra-task (*c* ∈ {*static, dynamic*}) difference in beta and gamma *IMC*_*area*_. A Wilcoxon signed rank test with BH correction was then used to determine the effect of the static and dynamic condition and the effect of timepoint on intra-task band difference in *IMC*_*area*_.


(5) $$\begin{array}{l} A_{c}=A_{coh}{\left(\gamma \right)}_{c}-A_{coh}{\left(\beta \right)}_{c} \end{array}$$

Finally, the correlation between *IMC*_*area*_ and stimulation intensity during static and dynamic trials with stimulation (TESCS_D) was considered by calculating Spearman's correlation coefficient.

## 3 Results

**Figure 3:**
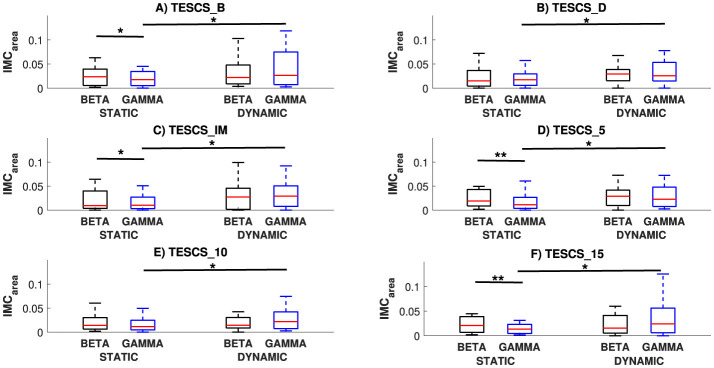
Comparison of *IMC*_*area*_ between conditions (static and dynamic) and frequency bands (beta and gamma) at each timepoint: before TESCS (TESCS_B) **(A)**; during TESCS, set to 80% tolerable intensity (TESCS_D) **(B)**; and immediately (TESCS_IM) **(C)**; 5 min (TESCS_5) **(D)**; 10 min (TESCS_10) **(E)**; and 15 min (TESCS_15) **(F)** after TESCS. Data is presented as median values with 25th and 75th percentiles. ^*^(*p* < 0.05) and ^**^(*p* < 0.01) denote statistically significant differences.

A comparison of *IMC*_*area*_ between different conditions and frequency bands at each timepoint is presented in Figure 3. This figure demonstrates the corticospinal network oscillates predominantly in the beta-band during the static task and gamma-band during the dynamic task. Comparing between the static and dynamic condition for the same frequency band across different timepoints, significantly greater gamma-band *IMC*_*area*_ was observed for the dynamic condition compared to the static condition at each timepoint (Figures 3A–F: *p*_*B*_ = 0.0183, *p*_*D*_ = 0.0183, *p*_*IM*_ = 0.0172, *p*_5_ = 0.0206, *p*_10_ = 0.0183, and *p*_15_ = 0.0172 respectively). There was no statistical significance between the static and dynamic condition for beta-band *IMC*_*area*_ (*p* > 0.05). Comparing between frequency bands for the same task across different timepoints, the static condition resulted in greater beta-band *IMC*_*area*_ whereas the dynamic condition showed no significant difference (*p* > 0.05), as shown in Figure 3. This increased beta- compared to gamma-band *IMC*_*area*_ for the static condition was statistically significant at TESCS_B (*p* = 0.0413, Figure 3A), TESCS_IM (*p* = 0.0447, Figure 3C), TESCS_5 (*p* = 0.0066, Figure 3D), and TESCS_15 (*p* = 0.0011, Figure 3F).

**Figure 4:**
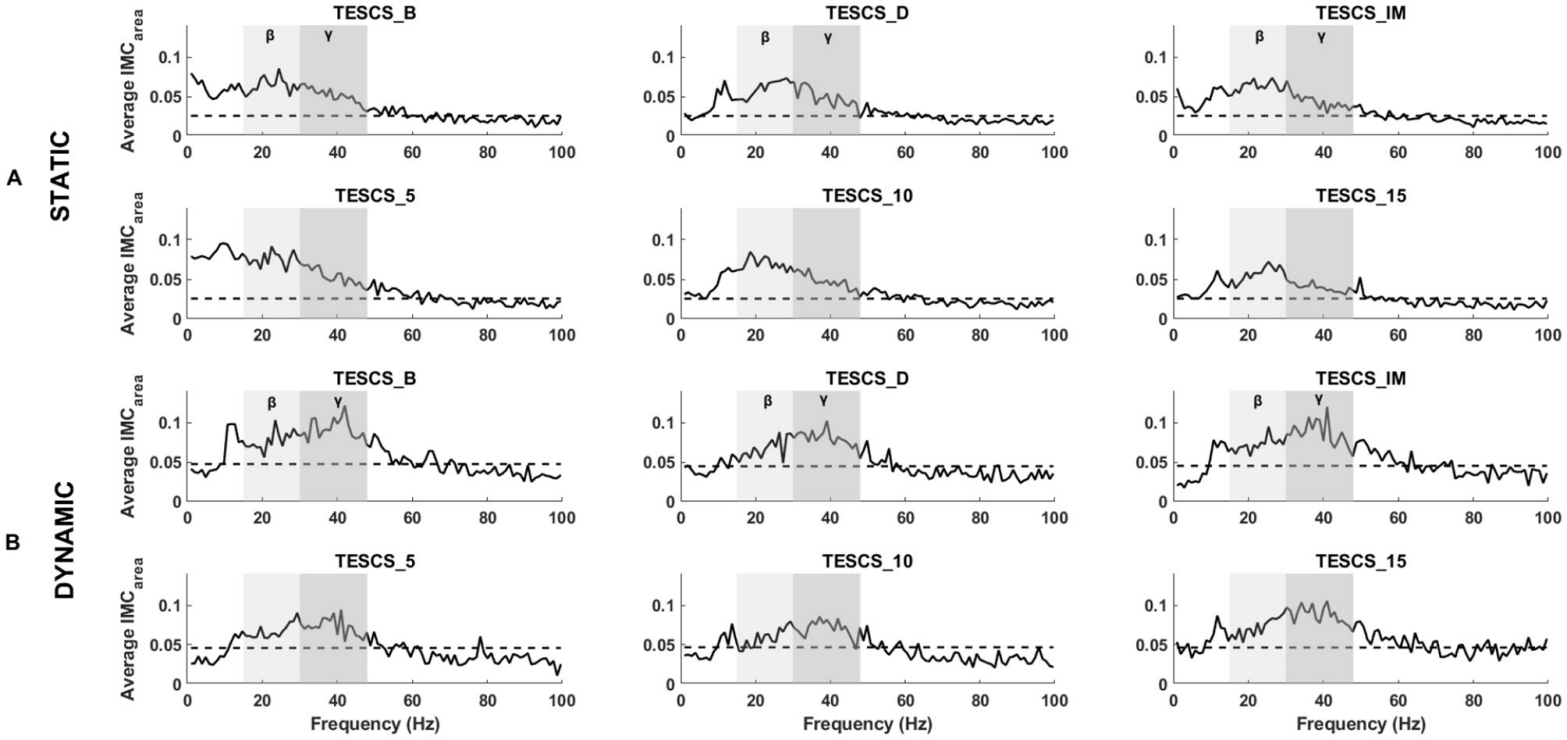
Grand averaged FDS-EDC *IMC*_*area*_ for the static **(A)** and dynamic **(B)** condition at each timepoint: before TESCS (TESCS_B); during TESCS, set to 80% tolerable intensity (TESCS_D); and immediately (TESCS_IM), 5 min (TESCS_5), 10 min (TESCS_10), and 15 min (TESCS_15) after TESCS. The beta frequency range is denoted by light gray shading and the gamma frequency range is shaded a darker gray. Horizontal dashed lines represent the 95% confidence limit above which coherence values are considered significant.

**Figure 5:**
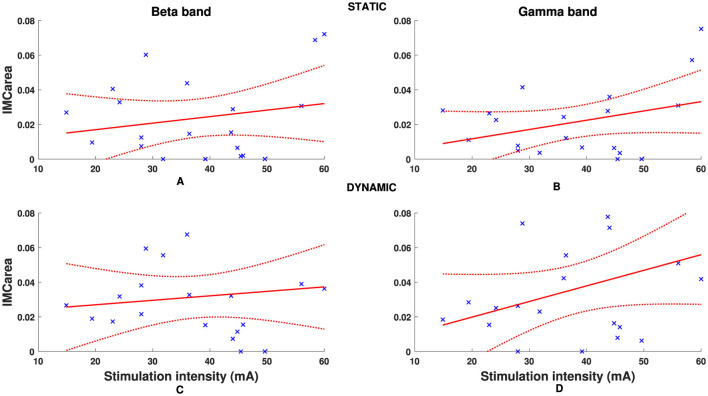
Individual participant FDS-EDC *IMC*_*area*_ in beta **(A, C)** and gamma **(B, D)** frequency bands for static **(A, B)** and dynamic **(C, D)** tasks. Dashed red line denotes 95% confidence intervals.

An overview of pooled *IMC*_*area*_ for all participants is presented in Figure 4, illustrating differences between static and dynamic conditions and frequency bands of interest. The timepoint at which the hand grip task was performed with respect to stimulation had no significant effect on *IMC*_*area*_ in the beta- or gamma-band for either the static (*p* = 0.25, *p* = 0.52) or dynamic condition (*p* = 0.31, *p* = 0.73) respectively. Although there was a positive correlation between stimulation intensity and *IMC*_*area*_, illustrated in Figure 5, this was not statistically significant in the beta- or gamma-band for either the static (*r*_β_ = 0.2111, *p*_β_ = 0.3715;*r*_γ_ = 0.3481, *p*_γ_ = 0.1326) or dynamic (*r*_β_ = 0.1326, *p*_β_ = 0.5774;*r*_γ_ = 0.3688, *p*_γ_ = 0.1096) condition respectively.

**Figure 6:**
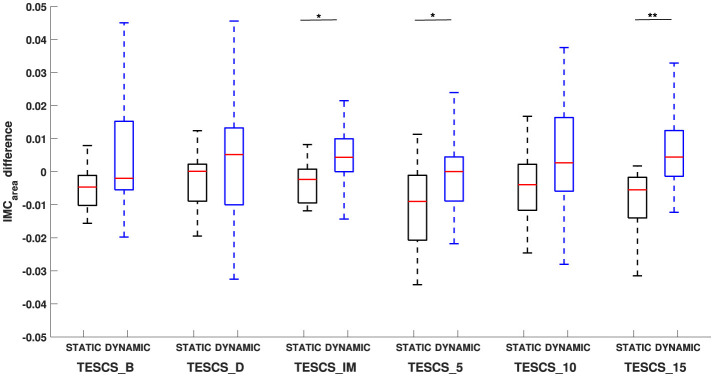
Comparison of the difference in beta-gamma *IMC*_*area*_ between static and dynamic conditions at each timepoint: before TESCS (TESCS_B); during TESCS, set to 80% tolerable intensity (TESCS_D); and immediately (TESCS_IM), 5 min (TESCS_5), 10 min (TESCS_10), and 15 min (TESCS_15) after TESCS. Positive values indicate greater *IMC*_*area*_ in the gamma-band and negative values indicate greater *IMC*_*area*_ in the beta-band. Data is presented as median values with 25th and 75th percentiles. ^*^(*p* < 0.05) and ^**^(*p* < 0.01) denote statistically significant differences.

Figures 4, 6 illustrate the shift in the prominence of *IMC*_*area*_ from beta to gamma frequencies between the static and dynamic condition. The intra-task difference in beta and gamma *IMC*_*area*_ between timepoints was not statistically significant (*p* > 0.05) for either the static or dynamic condition, as shown in Figure 6. However, the intra-task difference in beta and gamma *IMC*_*area*_ was significantly different between the static and dynamic condition after stimulation at TESCS_IM (*p* = 0.0275), TESCS_5 (*p* = 0.0275), and TESCS_15 (*p* = 0.0031).

## 4 Discussion

Both the static and dynamic hand grip task elicited significant IMC (above 95% CI) in both beta and gamma frequency bands. This aligns with a study that found tasks involving similar movements share common frequencies of coherence, suggested to result from common neural networks (Marsden et al., 2000). Although both tasks produced IMC in beta- and gamma-bands, the prominence of *IMC*_*area*_ in these frequency bands was determined by the type of task, with greater *IMC*_*area*_ in the beta-band compared to gamma-band during the static task. It is well documented that IMC is more prominent in the beta-band during constant force output which is thought to reflect cortical control of functionally related muscles (Kilner et al., 1999; Bravo-Esteban et al., 2014; Reyes et al., 2017). Indeed, studies have demonstrated beta-band IMC during co-contraction of antagonist muscle-pairs for static tasks (Hansen et al., 2002; Geertsen et al., 2013). Hansen et al. (2002) suggest IMC exhibited in the beta-band (15–30 Hz) during antagonist ankle muscle co-contraction is a result of common descending drive from the motor cortex by inhibiting pathways responsible for reciprocal inhibition, allowing synchronized discharge of corticospinal neurons to antagonist muscles. In agreement with this, Geertsen et al. (2013) attribute the increased IMC observed between antagonist ankle muscles to the suppression of inhibitory mechanisms, allowing greater activity of neural networks responsible for common drive to antagonist muscle-pairs. Although these studies do not compare beta and gamma frequency bands, they support the notion that co-contraction is mediated by common descending inputs. Results from our study support this, showing distinct coherence in the beta-band during the static condition which was significantly greater than the gamma-band. Thus, the increased IMC in this frequency band could be reflective of an increase in synchronized neural drive to antagonist forearm muscles during static hand grip.

The prominence of IMC has been shown to move from beta- to gamma-bands during dynamic muscle contraction (Omlor et al., 2007; Gwin and Ferris, 2012; Kenville et al., 2020). Omlor et al. (2007) attribute this shift in corticospinal oscillations to the demand for greater attentive resources and the rapid integration of this information required for a more complex motor output. The dynamic task implemented in the study by Omlor et al. required participants to isometrically track the periodically modulated force, demanding greater visual attention and more complex and continuous dynamic integration of visuomotor and somatosensory information than the static task. A later study by Gwin and Ferris (2012) expanded on this finding, suggesting the shift in frequency of coherence is influenced by muscle dynamics and proprioception rather than visuomotor and somatosensory information processing, as they observed a similar beta- to gamma- shift in coherence between isotonic and isometric tasks that did not differ in visual or sensory motor demands. Our results support these findings, as a distinct difference in the frequency of *IMC*_*area*_ between static and dynamic movement tasks was found with the dynamic task producing significantly greater gamma-band *IMC*_*area*_ compared to the static task across all conditions, including during stimulation (TESCS_D). Given that TESCS is believed to modulate spinal excitability and enhance sensorimotor connectivity, these findings have important implications for its application.

It would be intuitive to expect the dynamic task, due to the well-known antagonistic relationship of the EDC-FDS muscle pair, to exhibit less IMC. However, this would require participants to have actively extended their fingers, activating the EDC, during rest periods which participants were not instructed to do. This was demonstrated in a study by Motawar et al. (2016) investigating grip-relaxation, reporting FDS and EDC co-activation during maximum voluntary contraction trials and relaxation during rest periods when participants were instructed to release their grip without opening their fingers. Thus, the greater *IMC*_*area*_ produced by the dynamic task in the gamma frequency band could be reflective of increased integration of multi-sensory information, increasing corticomuscular drive. This could have implications for the type of movements that should be performed while receiving TESCS to enhance functional outcomes. Specifically, engaging in dynamic rather than static tasks during TESCS could facilitate stronger connectivity between the cortex and muscles, improving muscle coordination and functional recovery. Furthermore, supraspinal drive has been shown to be essential for transforming subthreshold sensory inputs generated by spinal cord stimulation into suprathreshold action potentials (Balaguer et al., 2023). Thus, the increase in corticospinal drive observed during dynamic tasks may enhance volitional motor neuron firing. These insights highlight the importance of task selection in movement-based rehabilitation protocols and are particularly relevant when incorporating TESCS, as dynamic tasks may inherently enhance functional outcomes.

Results from our study contrast with previous reports of reduced beta-band IMC from static to dynamic tasks (Omlor et al., 2007; Kilner et al., 1999). However, it has been suggested that this reduction in beta-band IMC only occurs during individual muscle control, highlighting that beta-band IMC reflects a synergistic control strategy that binds task-related motor neurons for coordinated motor output (Reyes et al., 2017). This was demonstrated in a study by Kenville et al. (2020) who reported a movement period-related modulation of common neural drive to homologous muscles during a compound movement task with significantly greater IMC in both beta- and gamma-bands during dynamic movement periods compared to static movement periods. Kenville et al. (2020) attributed the increase beta-band IMC to common cortical drive to functionally related muscles, since beta-band IMC has been shown to be more strongly influenced by the type of movement rather than somatosensory feedback (Nguyen et al., 2017). Our results indirectly support these findings, as the dynamic task required synergistic control which could be reflective of the lack of significant difference in beta-band *IMC*_*area*_ between static and dynamic tasks. This lack of significant change in beta-band *IMC*_*area*_ between tasks taken together with the significantly greater gamma-band *IMC*_*area*_ for the dynamic compared to static task, supports the notion that gamma-band IMC reflects a neural control strategy employed by the CNS during dynamic movement. This highlights the task-dependent frequency of common neural drive to muscles and provides further insight into the underlying mechanisms for sensorimotor control which can be used to inform movement-based therapies.

The results from this study show that TESCS does not significantly affect beta- or gamma-band *IMC*_*area*_ during static or dynamic tasks. Previous studies have shown that applying TESCS enables individuals with SCI to achieve greater movements, highlighting its potential to enhance the effectiveness of rehabilitation therapies (Moritz et al., 2024; Inanici et al., 2021, 2018). Therefore our finding is highly relevant as understanding the immediate effect of TESCS in able-bodied individuals is crucial for gaining insight into its underlying mechanisms and application in rehabilitation settings. The lack of immediate effect of TESCS on IMC could be explained by corticospinal networks already working at maximum capacity in able-bodied individuals, where the neural drive to muscles is already optimized for voluntary movement. Without corticospinal dysfunction, as seen in individuals with SCI, the addition of TESCS may not induce noticeable changes in IMC, since the system is functioning at its peak efficiency. However, further investigation into the intra-task difference in beta and gamma *IMC*_*area*_ between static and dynamic tasks revealed a significant difference only after stimulation. While greater gamma-band *IMC*_*area*_ was observed between static and dynamic tasks across all timepoints, the intra-task difference in frequency of corticospinal oscillations between the static and dynamic tasks was only significant after TESCS and not before or during. This supports the interpretation that TESCS could influence beta-gamma interactions during different handgrip tasks immediately post-intervention, although we have not demonstrated direct evidence for this. Thus, further investigation is needed to establish any causal effect.

## 5 Limitations

Although setting the intensity based on the participants' tolerance is common practice (McGeady et al., 2021, 2022; McHugh et al., 2020; Samejima et al., 2022; Gad et al., 2018), it presents the limitation that some participants may not reach an intensity high enough to adequately activate spinal neuronal networks. The intensity of stimulation should remain below motor threshold to ensure transsynaptic activation of α motor neurons via afferent fibers as opposed to direct stimulation of efferent pathways (Gerasimenko et al., 2015a; Novikov et al., 2024). However, intensities that are too low may not sufficiently depolarise group Ia afferent fibers. Conversely, intensities above motor threshold result in direct activation of efferent neurons, producing visible muscle twitches or involuntary movements (Gerasimenko et al., 2015a). To control for this, we ensured that stimulation remained below motor threshold, as evidenced by the absence of muscle twitching or visible movement. However, low intensities were not specifically controlled, which may have limited the activation of dorsal root afferents. This could be addressed by concomitant neurophysiological testing, such as posterior root muscle reflex post-activation depression, to confirm the activation of posterior roots by TESCS (Wu et al., 2020; Andrews et al., 2015). Although the results were not statistically significant, the positive correlation observed between stimulation intensity and *IMC*_*area*_ suggests a potential relationship that may have influenced the outcome of our study. It could be that the stimulation intensity was not strong enough to elicit a measurable effect in some participants. The study previously mentioned by Balaguer et al. (2023) on conventional implanted spinal cord stimulation highlighted the critical role of stimulation parameters in modulating motor neuron firing. The research demonstrated that a specific combination of frequency and current, in terms of Ia afferent recruitment, along with supraspinal input, facilitates excitatory postsynaptic potentials (Balaguer et al., 2023). This further highlights the importance of confirming dorsal root activation.

However, it is important to consider that results obtained by Balaguer et al. (2023)—from computational simulations, animal models, and individuals with neurological impairments, such as stroke or spinal cord injury - may not directly translate to our study on an able-bodied population using high-frequency non-invasive spinal cord stimulation. Further research is necessary to standardize stimulation parameters, and future studies should incorporate neurophysiological tests to confirm posterior root activation to deepen our understanding of the relationship between TESCS and IMC measurements.

This study was conducted on able-bodied individuals as a feasibility study to assess whether TESCS could influence IMC; however, no immediate effect was observed. One possible explanation is that the relatively short stimulation duration may have been insufficient to exert an immediate post-intervention measurable effect on *IMC*_*area*_. Previous research has demonstrated neuromodulatory effects in able-bodied individuals following 20 min of stimulation (Benavides et al., 2020), while IMC modulation in SCI individuals has been observed following 18 sessions of stimulation, applied for 15–45 min per session (McNicol et al., 2023). For this study, a shorter duration of TESCS was necessary to prevent carryover effects between conditions and to maintain consistent electrode positioning, as previously discussed. Despite this limitation, our results remain relevant in reference to the immediate effect of TESCS which has clinical applications in supporting rehabilitation therapy. Therefore, future work is encouraged to determine the critical duration of TESCS required to elicit an immediate measurable neuromodulatory effect post-intervention.

Another potential limitation is this study only recruited able-bodied participants therefore, results may be influenced by a ceiling effect whereby the common neural drive to muscles is already operating at maximum efficiency, therefore TESCS may not induce any measurable changes. As this was a preliminary feasibility study, the absence of an immediate TESCS effect does not necessarily indicate a lack of efficacy in individuals with impaired corticospinal function. Thus, this study should be repeated in a clinical population to better understand how TESCS influences common neural drive to regulate volitional motor control in those with impaired corticospinal function.

## 6 Conclusions

This study extends previous knowledge of task-dependent modulation of neural drive synchrony between antagonist forearm muscles. Comparison between two different types of tasks revealed the potential benefit of performing dynamic physical movements while receiving TESCS as opposed to static movements. Additionally, our findings provide insight into the effect of TESCS on descending motor control function. While our results suggest that TESCS may exert lasting neuromodulatory effects on the interaction of beta- and gamma-band IMC between static and dynamic tasks, direct evidence to confirm this was not demonstrated. Furthermore, this study indicates that it is not feasible to immediately enhance the task-dependent frequency of IMC with TESCS in able-bodied individuals. Given TESCS has shown the ability to modulate IMC in a clinical population, it may be that TESCS has limited ability to modulate the spinal circuitry of able-bodied individuals or that the duration of TESCS was insufficient to elicit an effect in able-bodied participants. Future research using coherence measures are encouraged to further understand the effect of TESCS-based rehabilitation therapy on common descending neural drive.

## Data Availability

The raw data supporting the conclusions of this article will be made available by the authors, without undue reservation.
